# Improving the Health and Well-Being of Individuals by Addressing Social, Economic, and Health Inequities (Healthy Eating Active Living): Protocol for a Cohort Study

**DOI:** 10.2196/41169

**Published:** 2025-02-13

**Authors:** Ashish Joshi, Surapaneni Krishna Mohan, Apurva Kumar Pandya, Ashoo Grover, Harpreet Kaur, Mansi Gupta, Heemanshu Aurora, Ashruti Bhatt

**Affiliations:** 1 School of Public Health, University of Memphis Memphis, TN United States; 2 Panimalar Medical College Hospital & Research Institute Chennai India; 3 Parul Institute of Public Health Vadodara, Gujarat India; 4 Indian Council of Medical Research New Delhi India; 5 Foundation of Healthcare Technologies Society New Delhi India

**Keywords:** health inequity, health, well-being, digital interventions, social health, lifestyle

## Abstract

**Background:**

Health inequity is interlinked with the good health and well-being of an individual. Health inequity can be due to various socioeconomic factors like income levels or social status. Digital health interventions have the potential to reduce the existing health inequities.

**Objective:**

This study aims to identify determinants of social, economic, and health inequity in diverse settings to enhance healthy eating and active living. It further aims to design and develop a digital health intervention HEAL (Healthy Eating Active Living) that incorporates a human-centered design framework in order to improve healthy eating and active living among rural and urban population groups in Chennai, Tamil Nadu, India.

**Methods:**

A prospective, 3-year cohort study will be conducted. This study aims to recruit 6350 individuals across rural and urban settings of Chennai. A total of 11 sites have been selected for participation in the study. Data on sociodemographic characteristics; economic inequity; HEAL profile; depression, anxiety, and stress; well-being; sources of health information; perceived access to health care; health literacy; navigation of health literacy; and satisfaction with the health system will be gathered. This study would help to explore the determinants of social, economic, and health inequity across multiple sites. SAS (version 9.3; SAS Institute Inc) will be used for data analysis, and results will be reported as 95% CI and *P* values. This study’s findings will guide the design and development of a tailored, human-centered digital health intervention to enhance the health and well-being of Chennai’s population groups.

**Results:**

As of December 2024, the literature review for the development of the intervention has been completed. The recruitment for the baseline data collection will begin shortly, followed by the development of HEAL intervention.

**Conclusions:**

The proposed study will help in examining the role of the proposed HEAL intervention to enhance the health and well-being of the population groups of Chennai.

**International Registered Report Identifier (IRRID):**

PRR1-10.2196/41169

## Introduction

### Background and Rationale

The World Health Organization (WHO) identifies health inequities as “systematic differences in the health status of different population groups” [1]. Further, the WHO refers to health as “a state of complete physical, mental, and social well-being and not merely the absence of disease or infirmity” [2]. Consequently, research on health inequity is a central agenda component of the 2030 Sustainable Development Goals and various government and development dialogues [3,4]. Despite this, the WHO reports that health inequalities exist at varying degrees around the world, both within and between countries [5]. These inequalities further exacerbate health disparities between high-income and low-income, urban and rural, employed and unemployed populations, as well as between low-, middle-, and high-income countries [6] due to socioeconomic factors such as social status or income levels [7].

According to the Centers for Disease Control and Prevention, health equity can be attained when every individual can “attain his or her full health potential” and no one is “disadvantaged from achieving this potential because of social position or other socially determined circumstances” [8]. This also emphasizes the significance of measuring health inequity, promoting opportunities and monitoring the progress in health and intersectoral strategies. Additionally, measuring the interrelationship between health and its social determinants is vital to promoting effective interventions for health equity [9].

Inequalities are unfair, affect everyone, and are avoidable; however, the underlying factors causing inequality are imperfectly understood, and evidence on the effectiveness of interventions to reduce inequalities is limited [10]. Prior research has further highlighted the issue of good health for socioeconomically vulnerable groups and the need for targeted public health interventions in India [11]. A study indicated that health inequity in India is favored toward high-income populations and that despite services being available to people, there was a lack of awareness around it, especially in the rural region [12]. Considering the diversity in India and the prevalent social inequalities, the literature also emphasizes on the importance of research on health inequalities for monitoring health policies and programs [11].

Equity was also identified as one of the key principles of India’s 2017 National Health Policy [13]. However, with a limited evidence base of health equity in India, there is a need to generate empirical data for the variables that create, sustain, and reinforce inequity. This would further help the policy makers to prioritize and strategize the limited resources available for the health system of the country [14].

However, the paucity of data on health inequalities in India and the subsequent research gap on critical issues related to health inequity in the country also underscore the probable impact of policies and strategies deployed to enhance the well-being of population groups. Moreover, the COVID-19 pandemic has exacerbated the health inequity worldwide [15]. In addition, eating habits are possibly determined by the socioeconomic status of the individuals [16]. Thus, this study aims to enrich the current understanding of health inequity with empirical research. The findings will also help with the design and development of digital health intervention HEAL (Healthy Eating Active Living) to improve the good health and well-being of individuals by addressing social, economic, and health inequities. Tamil Nadu, India, was the chosen region of study as it seems to be a state that has progressed in health care in recent years, and hence, this study aims to see the receptivity to digital intervention of the study population in these settings [12].

### Study Objectives

This study aims (1) to identify determinants of social, economic, and health inequity to enhance healthy eating and active living among rural and urban population groups of Chennai, Tamil Nadu; (2) to design and develop a digital health intervention HEAL using the SMAART (Sustainable, Multi-Sector, Accessible, Affordable, Reimbursable, Tailored) model, targeted to enhance healthy eating and active living among rural and urban population groups of Chennai, Tamil Nadu; and (3) to evaluate impact of the proposed intervention to address burden of social, economic, and health inequity to enhance healthy eating and active living among rural and urban population groups of Chennai, Tamil Nadu.

## Methods

### Study Overview

The proposed research involves a multidisciplinary team with expertise in population health, epidemiology, sociobehavioral sciences, anthropology, and biochemical sciences.

### Study Design and Population

The HEAL cohort study is a prospective, 3-year cohort study with the aim to recruit 6350 individuals across rural and urban settings of Chennai, Tamil Nadu. The study sample will be recruited from Kannur; Pannur; Thirumenikuppam; Thodukadu; Kottaiyur; Nemili; Mannur; Kiloy; Ulundai; Narasamangalam; Karanai; Panimalar Medical College Hospital and Research Institute; Rural Training Health Centre Chennai; and Maligaipaddu village of Chennai, Tamil Nadu. Baseline evaluation will be performed at the time of recruitment of study participants. The study participants will be recruited through convenience sampling. This will serve as cross-sectional data to estimate the prevalence of determinants of social, economic, and health inequity.

### Study Intervention

The HEAL intervention will be designed based on the SMAART model to enhance healthy eating and active living for the individuals participating in the study. This intervention will ensure the coordination of social, economic, and health-related parameters to enhance the health and well-being status of the population. The SMAART model works on the principles of data, information, and knowledge; human-centered approach; information processing theory; and humanistic, behavioral, and learning theories [17]. The intervention stage will have two parallel groups (case and control) for comparison. Individuals in the intervention group will be provided access to the informatics-based intervention for enhancing their healthy eating and active living. The participants from the intervention stage will be included in the study based on participants from the baseline stage consenting to participate in the intervention. The participants will be assigned to the intervention or control group through randomization. They would be provided information on good dietary habits and the importance of regular physical exercise for maintaining good health and well-being. Individuals in the control group will be provided an educational booklet as standard care. The intervention will be a tailored multilingual digital health intervention. Both the groups will undergo a series of baseline and follow-up assessments for estimation of change in eating patterns, physical activity, and well-being to assess the impact of intervention. The study location includes both the urban and rural areas of selected sites, and the study duration is 3 years. The study population includes all adults aged 18 years and older. The inclusion criteria are (1) individuals aged 18 years and older, (2) individuals from both urban and rural areas, and (3) individuals who consent to participate in the study.

Efforts will be made to include individuals of varied age groups, gender, location, and socioeconomic status. The exclusion criteria consist of (1) individuals younger than 18 years of age, (2) individuals who would not give their consent, and (3) individuals who have impaired cognition.

### Ethical Considerations

This study (protocol PMCHRI-IHEC-067) gained approval from the Panimalar Medical College Hospital and Research Institute Institutional Human Ethics Committee (Central Drugs Standard Control Organization Registration ECR/1399/Inst/TN/2020) in March 2022, with approval PMCH&RI/IHEC/2021/078 dated March 15, 2022. The study will be conducted according to the Declaration of Helsinki, as it involves human participants [18].

The institutional review board–approved informed consent form will be administered by the research team to the eligible individuals for the study. The research team will describe the study, time required, and benefits of the study results to the participants, and those willing to participate and give their consent will be enrolled in the study. Written informed consent will be obtained in both English and local Indian dialects at the time of data collection if the participants are able to read and understand the questionnaire. In the case of participants without formal education, an audio recording of the consent will be done. Study participants will be allowed to withdraw from the study at any time [19]. No compensation will be provided.

Data security will be ensured through regular backups in password-protected computers in the locked office of the principal investigator. Data will be stored for 5 years from the point of study completion, after which it will be destroyed. All the physical data files will be stored in a locked file cabinet in the office. The information will be accessible to members of the research team only.

### Data Collection, Data Entry, and Quality Assurance

Data collection and data entry will be performed by a team of trained field data collectors and data management. Data will be gathered in the field on paper and then entered on the computer. The data will be entered into the computer using Excel (Microsoft Corp). For quality assurance, a clearly defined data collection and management protocol will be in place. This will include a well-defined study manual with all the relevant instructions. For quality assurance, we will have a trained team of data collectors, weekly meetings with the research team, weekly data checks, maintenance of study participant contact, and maintenance of study participant data instrument logs.

### Variable Assessment

#### Sociodemographic Characteristics

Information will be collected on the participant’s age, gender, educational status, migration status, disability status, occupation, household size, family size, income, and number of earning members in the household.

#### Economic Inequity

The wealth index is a division of households into 5 wealth quintiles to show the relationship between wealth, population, and health indicators [20].

#### HEAL Profile

Data will be collected on the respondent’s health and lifestyle profile. We will use the WHO STEPwise approach to noncommunicable diseases risk factor surveillance questionnaire. It is a simple, standardized method for collecting, analyzing, and disseminating data on key noncommunicable disease risk factors in countries. The survey instrument covers key behavioral risk factors: tobacco use, alcohol use, physical inactivity, and unhealthy diet, as well as key biological risk factors: overweight and obesity, raised blood pressure, raised blood glucose, etc [21].

#### Depression, Anxiety, and Stress

Data will be gathered from the respondents about their mental and well-being. We will use the Depression, Anxiety, and Stress Scale (DASS). The DASS is a set of three self-report scales designed to measure the negative emotional states of depression, anxiety, and stress. Each of the three DASS scales contains 14 items, divided into subscales of 2-5 items with similar content [22].

#### Well-Being

The Short Warwick Edinburgh Mental Well-Being Scale is a shortened version of the Warwick-Edinburgh Mental Well-Being Scale. It measures both mental and emotional well-being (how “good” somebody feels) and psychological functioning (how well somebody thinks they are functioning). This scale is suitable for ages 13-74 years and works well as a “before” and “after” tool to measure the change in well-being during an intervention or specific program [23].

#### Sources of Health Information, Social Media, Technology Access, and Familiarity

Information will be gathered about cell phone ownership in households, type of cell phone, access to the internet, and knowledge of SMS text messaging [24,25].

#### Perceived Access to Health Care

Information will be collected from the respondents regarding access to health care. We will use the Perceived Access to Health Care questionnaire. The questionnaire includes six dimensions: (1) availability, (2) accessibility, (3) affordability, (4) accommodation, (5) acceptability, and (6) awareness. In addition, the psychometric evaluation was conducted on the instrument’s initial version with 31 items on a 5-point Likert scale (strongly agree to strongly disagree) [26].

#### Health Literacy

Rapid Estimate of Adult Literacy in Medicine will be used to assess the health literacy of the study participants. It is a quick screening tool to assist physicians in identifying patients with limited reading skills and in estimating patient reading levels. “At risk patients” are defined as those with a score of six or less [27].

#### Satisfaction With Health System

The Patient Satisfaction Questionnaire yields separate scores for each of seven different subscales: general satisfaction (2 items), technical quality (4 items), interpersonal manner (2 items), communication (2 items), financial aspects (2 items), time spent with the doctor (2 items), and accessibility and convenience (4 items). This questionnaire will be used both at baseline and postintervention [28,29].

### Outcomes

The study outcomes include the identification of determinants of social, economic, and health inequity. The research would further help in identifying the key areas that need to be addressed through intervention to improve the health and well-being status of the general population. Additionally, the study would also help in developing tailored interventions targeted to reduce social, economic, and health inequities.

### Data Analysis

Descriptive analysis will be conducted to report the mean and SD of the continuous variables and frequency analysis of the categorical variables. A 1-tailed *t* test will be performed to compare means between the continuous variables and a categorical dependent variable while a chi-square analysis will be performed for the categorical variables. Multivariate regression analysis will be performed to determine the determinants of social, economic, and health inequity. All analysis will be performed using SAS (version 9.1; SAS Institute Inc), and reporting of the results will be done at 95% CI and *P*<.05.

### Projected Timelines and Milestones

A detailed research plan and scheduled timeline of the tasks involved in the study are presented in Table 1.

**Table 1:** Scheduled timeline of the tasks in the HEAL^a^ study.

Variables	Month 1	Months 2-5	Months 6-7	Months 8-10	Months 11-18	Months 14-30	Months 31-33	Months 34-36
Stakeholder meeting	✓							
Baseline data collection		✓						
Design and development of human-centered intervention			✓					
Heuristic evaluation			✓					
Usability of the proposed system				✓				
Refine the proposed SMAART^b^ informatics self-management intervention				✓				
Final deployment of the proposed SMAART informatics self-management intervention tool				✓				
Study recruitment (baseline data)					✓			
Follow-up data collection					✓	✓		
Statistical analysis					✓	✓	✓	
Report writing and paper preparation							✓	✓

^a^HEAL: Healthy Eating Active Living.

^b^SMAART: Sustainable, Multi-Sector, Accessible, Affordable, Reimbursable, Tailored.

The study is in accordance with the STROBE (Strengthening the Reporting of Observational Studies in Epidemiology) guideline for case-control studies (Multimedia Appendix 1) [30].

## Results

As of December 2024, the literature review for the development of the intervention has been completed. Recruitment for the baseline data collection is scheduled to begin in the upcoming months, followed by the development of HEAL intervention.

The findings of the study will be disseminated through peer-reviewed publications and national and international conference presentations.

## Discussion

### Expected Findings

In order to reduce the health inequity in India, it is imperative to have the requisite knowledge base. This study would help to fill the research and knowledge gap by identifying key areas that need to be addressed to improve the health status of the population. The baseline data will enable us to find the socioeconomic inequity faced by the participants, their health literacy, and their awareness regarding their health status and health care. These findings would also help to design and develop tailored population health interventions to enhance the healthy eating and active living of the population groups.

Health inequities have existed throughout time, as resonated by the concept of inverse care law (1971), which highlighted how good medical care is inversely related to people’s needs in low- and middle-income countries [31]. Similarly, Cookson et al [32] recently proposed the law of disproportionate care (2021) where they argue that in high-income countries, socially disadvantaged groups receive more care but of worse quality to meet their health care needs. The available studies also reinstate the need to address health inequities by collecting and analyzing the relevant health equity data [33]. This study would therefore help in filling the requisite knowledge gaps by identifying the key determinants of health inequity. It would also help in contributing to the advancement of evidence-based strategic decisions on health inequity. The findings from this research project would also help in designing, developing, and implementing data-driven, evidence-based, and human-centered interventions to enhance the healthy eating and active living of the population groups.

### Limitations

This study’s limitations include that it is confined to one geographic region, and implementing it in diverse geographic and population settings would help to better understand and compare the impact of the HEAL intervention.

### Conclusions

The HEAL intervention offers a model to enhance healthy eating and active living for the individuals participating in the study through the SMAART framework. The findings from the study will contribute to the understanding of the role of digital health interventions to enhance healthy eating and active living of the population groups, which will help in understanding and addressing determinants of health equity in the population.

## Appendix

Multimedia Appendix 1 STROBE (Strengthening the Reporting of Observational Studies in Epidemiology) checklist.

## Abbreviations

DASS: Depression Anxiety, and Stress Scale
HEAL: Healthy Eating Active Living
SMAART: Sustainable, Multi-Sector, Accessible, Affordable, Reimbursable, Tailored
STROBE: Strengthening the Reporting of Observational Studies in Epidemiology
WHO: World Health Organization

## Data Availability

The datasets generated during or analyzed during this study will be available from the corresponding author upon reasonable request.
