# Supplementary material for: Improving the Health and Well-Being of Individuals by Addressing Social, Economic, and Health Inequities (Healthy Eating Active Living): Protocol for a Cohort Study
Source: JMIR Res Protoc. 2025 Feb 13;14:e41169. doi: 10.2196/41169 (PMC11888054; doi:10.2196/41169)
Supplement: Multimedia Appendix 1 [file resprot_v14i1e41169_app1.docx]

STROBE Statement—Checklist of items that should be included in reports of ***case-control studies***

|  | | Item No | Recommendation | Page No | Line No. |
| --- | --- | --- | --- | --- | --- |
| **Title and abstract** | | 1 | (*a*) Indicate the study’s design with a commonly used term in the title or the abstract | 1 | 3, 20 |
|  |  |  | (*b*) Provide in the abstract an informative and balanced summary of what was done and what was found | 1 | 11-42 |
| Introduction | | | | | |
| Background/rationale | | 2 | Explain the scientific background and rationale for the investigation being reported | 2,3 | 69-80 |
| Objectives | | 3 | State specific objectives, including any prespecified hypotheses | 3 | 81-89 |
| Methods | | | | | |
| Study design | | 4 | Present key elements of study design early in the paper | 3,4 | 94-123 |
| Setting | | 5 | Describe the setting, locations, and relevant dates, including periods of recruitment, exposure, follow-up, and data collection | 4,5 | 124-125, 221-224 |
| Participants | | 6 | (*a*) Give the eligibility criteria, and the sources and methods of case ascertainment and control selection. Give the rationale for the choice of cases and controls | 4 | 124-135 |
|  |  |  | (*b*) For matched studies, give matching criteria and the number of controls per case | NA |  |
| Variables | | 7 | Clearly define all outcomes, exposures, predictors, potential confounders, and effect modifiers. Give diagnostic criteria, if applicable | 5 | 163-205 |
| Data sources/ measurement | | 8* | For each variable of interest, give sources of data and details of methods of assessment (measurement). Describe comparability of assessment methods if there is more than one group | NA |  |
| Bias | | 9 | Describe any efforts to address potential sources of bias | NA |  |
| Study size | | 10 | Explain how the study size was arrived at | NA |  |
| Quantitative variables | | 11 | Explain how quantitative variables were handled in the analyses. If applicable, describe which groupings were chosen and why | 5 | \163-205 |
| Statistical methods | | 12 | (*a*) Describe all statistical methods, including those used to control for confounding | 5 | 213-220 |
|  |  |  | (*b*) Describe any methods used to examine subgroups and interactions | 5 | 213-220 |
|  |  |  | (*c*) Explain how missing data were addressed | 5 | 213-220 |
|  |  |  | (*d*) If applicable, explain how matching of cases and controls was addressed | 5 | 213-220 |
|  |  |  | (*e*) Describe any sensitivity analyses | 5 | 213-220 |
| Results | | | | | |
| Participants | | 13* | (a) Report numbers of individuals at each stage of study—eg numbers potentially eligible, examined for eligibility, confirmed eligible, included in the study, completing follow-up, and analysed | NA |  |
|  |  |  | (b) Give reasons for non-participation at each stage | NA |  |
|  |  |  | (c) Consider use of a flow diagram | NA |  |
| Descriptive data | | 14* | (a) Give characteristics of study participants (eg demographic, clinical, social) and information on exposures and potential confounders | NA |  |
|  |  |  | (b) Indicate number of participants with missing data for each variable of interest | NA |  |
| Outcome data | | 15* | Report numbers in each exposure category, or summary measures of exposure | NA |  |
| Main results | | 16 | (*a*) Give unadjusted estimates and, if applicable, confounder-adjusted estimates and their precision (eg, 95% confidence interval). Make clear which confounders were adjusted for and why they were included | NA | |
|  |  |  | (*b*) Report category boundaries when continuous variables were categorized | NA | |
|  |  |  | (*c*) If relevant, consider translating estimates of relative risk into absolute risk for a meaningful time period | NA | |
| Other analyses | 17 | Report other analyses done—eg analyses of subgroups and interactions, and sensitivity analyses | | NA | |
| Discussion | | | | | |
| Key results | 18 | Summarise key results with reference to study objectives | | NA | |
| Limitations | 19 | Discuss limitations of the study, taking into account sources of potential bias or imprecision. Discuss both direction and magnitude of any potential bias | | NA | |
| Interpretation | 20 | Give a cautious overall interpretation of results considering objectives, limitations, multiplicity of analyses, results from similar studies, and other relevant evidence | | NA | |
| Generalisability | 21 | Discuss the generalisability (external validity) of the study results | | NA | |
| Other information | | | | | |
| Funding | 22 | Give the source of funding and the role of the funders for the present study and, if applicable, for the original study on which the present article is based | | NA | |

*NA- Not applicable
